# Phenotypes and Genotypes of Children with Vitamin D-Dependent Rickets Type 1A: A Single Tertiary Pediatric Center in Vietnam

**DOI:** 10.3390/diagnostics15070918

**Published:** 2025-04-02

**Authors:** Thi Anh Thuong Tran, Tran Minh Dien, Ngoc Lan Nguyen, Khanh Ngoc Nguyen, Thi Bich Ngoc Can, Bui Phuong Thao, Nguyen Thi Thuy Hong, Van Khanh Tran, Thinh Huy Tran, Ngo Xuan Khoa, Nguyen Thi Kim Lien, Nguyen Thien Tao, Huy Hoang Nguyen, Chi Dung Vu

**Affiliations:** 1 Department of Paediatrics, Hanoi Medical University, Hanoi 11521, Vietnam; trananhthuong@hmu.edu.vn (T.A.T.T.); khanhnn@nch.gov.vn (K.N.N.); bshong@hmu.edu.vn (N.T.T.H.); 2 Center of Endocrinology, Metabolism, Genetic/Genomics and Molecular Therapy, Vietnam National Children’s Hospital, Hanoi 11512, Vietnam; ngocctb@nch.gov.vn (T.B.N.C.); thaobp@nch.gov.vn (B.P.T.); 3 Vietnam National Children’s Hospital, Hanoi 11512, Vietnam; dientm@nch.gov.vn; 4 Center for Gene and Protein Research, Hanoi Medical University, Hanoi 11521, Vietnam; nguyenngoclan@hmu.edu.vn (N.L.N.); tranvankhanh@hmu.edu.vn (V.K.T.); tranhuythinh@hmu.edu.vn (T.H.T.); ngoxuankhoavn@gmail.com (N.X.K.); 5 Institute of Genome Research, Vietnam Academy of Science and Technology, Hanoi 100000, Vietnam; ntkimlienibt@gmail.com (N.T.K.L.); nguyenthientao@igr.ac.vn (N.T.T.)

**Keywords:** vitamin D-dependent rickets type 1A, *CYP27B1*, Vietnamese children, skeletal deformities, ricket, hypocalcemia, elevated alkaline phosphatase, elevated parathyroid hormone

## Abstract

**Background**: Vitamin D-dependent rickets type 1A (VDDR1A) is a rare autosomal recessive disorder caused by mutations in the *CYP27B1* gene, leading to a deficiency in active vitamin D (1,25-dihydroxyvitamin D). This study examines the genotypic and phenotypic characteristics of VDDR1A in Vietnamese children. **Patients and Methods**: A retrospective analysis was conducted on 19 Vietnamese children diagnosed with VDDR1A. Clinical, radiological, biochemical, and molecular data were collected. Rickets Severity Scores (RSSs), biochemical parameters, and height standard deviation scores (HtSDSs) were used to assess the severity of the condition. **Results:** The study included 19 children from 17 families (ten males and nine females). The median age of rickets diagnosis was 19.2 months, while with VDDR1A, the median time of diagnosis was 7.5 months. Common symptoms among the children included thickened wrists and ankles (19/19), genu varum or genu valgum (18/19), failure to thrive (18/19), rachitic rosary (12/19), and delayed walking (11/19). The radiographic features showed that all children had cupping, splaying, and fraying, twelve children had rachitic rosary, and six exhibited pseudofractures. The biochemical findings showed severe hypocalcemia, normal or mildly low serum phosphate, elevated alkaline phosphatase and parathyroid hormone levels, and normal serum 25-hydroxyvitamin D levels. Genetic analysis identified biallelic *CYP27B1* variants, including one known pathogenic frameshift mutation, c.1319_1325dup p.(Phe443Profs*24), one novel likely pathogenic missense variant, c.616C>T p.(Arg206Cys), and one novel pathogenic frameshift mutation, c.96_97del p.(Ala33Thrfs*299). The c.1319_1325dup p.(Phe443Profs*24) variant was the most common, present in 18 out of 19 children. **Conclusions:** The children with VDDR1A in this study presented with growth failure and skeletal deformities. Key findings included severe hypocalcemia, elevated alkaline phosphatase and parathyroid hormone levels, normal or elevated 25(OH)D, and high RSSs. Predominant frameshift mutations in *CYP27B1*, especially c.1319_1325dup, highlighted the importance of early genetic diagnosis for optimal management. Additionally, two novel *CYP27B1* variants were identified, expanding the known mutation spectrum of VDDR1A.

## 1. Introduction

Rickets was first described in the 17th century by Whistler (1645) and Glisson (1650) [1]. Later, Albright et al. (1937) provided a detailed definition of vitamin D-resistant rickets [2]. In 1961, Prader first reported vitamin D-dependent rickets (VDDR) [3]. VDDR is classified into five types or subtypes: VDDR type 1A (VDDR1A, MIM # 26470), VDDR type 1B (VDDR1B, MIM # 600081), VDDR type 2A (VDDR2A; MIM # 277440), VDDR type 2B (VDDR2B, MIM # 600785), and VDDR type 3 (VDDR3, MIM # 619073) [4].

Vitamin D-dependent rickets type 1A (VDDR1A) is a rare autosomal recessive disorder caused by biallelic mutations in the *CYP27B1* gene (MIM * 609506), which encodes the enzyme 1-alpha hydroxylase [5]. The *CYP27B1* gene is located on chromosome 12p13.3 and consists of nine exons. This enzyme is critical for converting 25-hydroxyvitamin D (25(OH)D) into its active form, 1,25-dihydroxyvitamin D (calcitriol), in the kidneys. Calcitriol regulates calcium and phosphate homeostasis, promoting normal bone mineralization and skeletal development. A deficiency or dysfunction of the CYP27B1 enzyme leads to severe hypocalcemia, hypophosphatemia, and impaired bone mineralization [6]. The global prevalence of VDDR1A is not accurately known. In Denmark, the prevalence of hereditary rickets is estimated at 4.8 per 100,000 children [7]. In the Charlevoix–Saguenay–Lac-Saint-Jean region of Québec, De Braekeleer and Larochelle estimated the prevalence of VDDR1A to be one in 2916 live births, with a carrier rate of one in 27 individuals [8].

Vitamin D-dependent rickets type 1A (VDDR1A) is characterized by skeletal deformities such as bowed legs (genu varum), knock-knees (genu valgum), thickened wrists and ankles, frontal bossing, hypotonia, failure to thrive, seizures, and delayed motor milestones [9]. In severe cases, hypocalcemia-induced seizures may occur [9,10,11,12]. Growth retardation is a prominent feature of VDDR1A, as impaired bone mineralization delays bone formation and growth plate closure, leading to shorter stature and slower skeletal development, significantly impacting final height [4].

In VDDR1A, calcium levels are typically low due to the impaired intestinal absorption of calcium caused by the deficiency in active vitamin D [13]. Phosphate levels may initially be within the normal or low-normal range, but generally decline over time due to prolonged parathyroid hormone (PTH)-induced renal phosphate wasting [14]. Elevated alkaline phosphatase (ALP) levels are a hallmark of VDDR1A, reflecting increased osteoblastic activity as the body attempts to compensate for impaired bone mineralization [15]. The persistent elevation of PTH levels indicates secondary hyperparathyroidism, a compensatory response to hypocalcemia. Vitamin D metabolism is significantly disrupted in VDDR1A, with the 25-hydroxyvitamin D [25(OH)D] levels often appearing normal or mildly elevated, further complicating the biochemical profile of this disease [9].

Radiographic imaging is crucial in diagnosing VDDR1A [4,9,16]. Early radiographic signs include an increased growth plate height, the widening of the epiphyseal plate, and the disappearance of the zone of provisional calcification at the interface between the epiphysis and the metaphysis, reflecting delayed and disrupted mineralization [4]. Distinctive features, such as cupping, fraying, and splaying of the metaphyses, are typically observed, particularly in the wrists, knees, and ankles [16]. Additionally, pseudofractures on the compression side of the bone, known as Looser’s zones, have been noted [9].

The *CYP27B1* pathogenic variants causing VDDR1A are diverse, including missense, nonsense, frameshift, and splice site mutations [17]. According to the Human Gene Mutation Database (HGMD) (https://www.hgmd.cf.ac.uk/ac/gene.php?gene=CYP27B1; accessed on 7 February 2025), a total of 116 pathogenic mutations in the *CYP27B1* gene have been identified as being associated with VDDR1A. The most commonly reported mutations across different populations with VDDR1A are p.F443Pfs*24, c.195+2T>G, and p.V88Wfs*71 [17]. These mutations occur in either homozygous or compound heterozygous states, contributing to the genetic diversity observed in VDDR1A cases.

In Vietnam, studies on hereditary rickets, including VDDR1A, are limited. Although rickets is a well-documented health concern among Vietnamese children, most cases are attributed to nutritional deficiencies due to inadequate vitamin D intake and limited sunlight exposure. This study aims to describe the clinical, radiological, biochemical, and molecular characteristics of 19 Vietnamese children from 17 unrelated families diagnosed with VDDR1A at the Vietnam National Children’s Hospital between January 2023 and December 2024.

## 2. Materials and Methods

### 2.1. Subjects

This retrospective descriptive study focused on children with a confirmed diagnosis of VDDR1A. Data were collected from medical records, including clinical, biochemical, radiological, and genetic information. A total of 19 children from 17 families were included in the study.

The children were diagnosed with VDDR1A based on clinical signs, biochemical findings, radiological features, and genetic testing. The clinical signs included skeletal deformities such as genu varum, genu valgum, rachitic rosary, and frontal bossing. The biochemical studies revealed hypocalcemia, normal or low serum phosphate, and elevated serum levels of alkaline phosphatase and parathyroid hormone, with normal or increased plasma concentrations of 25(OH)D. The radiological findings showed cupping, splaying, fraying, rachitic rosary, or pseudofractures on the X-rays. The genetic testing confirmed the diagnosis by identifying the biallelic pathogenic variants in the *CYP27B1* gene.

The study was conducted at the Center for Endocrinology, Metabolism, Genetics, and Molecular Therapy at the Vietnam National Children’s Hospital from January 2023 to December 2024.

### 2.2. Clinical Characteristics

Data were extracted from medical records and laboratory databases. Height and weight were evaluated using the WHO growth chart. Biochemical parameters including serum calcium, serum phosphate, alkaline phosphatase (ALP), and parathyroid hormone (PTH) were measured using standardized methods on the Beckman Coulter AU5800 (Beckman Coulter, Tokyo, Japan) at the biochemistry department. Bone X-rays were performed using the Carestream DRX1-System (Carestream, WA, USA) at the diagnostic imaging department. Radiographs of the wrists and knees were examined for signs of rickets. To quantify the severity of the radiographic changes, the Rickets Severity Score (RSS) was applied [16]. This scoring system evaluates abnormalities at key skeletal sites, including the wrists and knees, based on growth plate widening, metaphyseal fraying, and cupping (Table 1). Each parameter is scored on a scale from 0 (normal) to 10 (severe), with higher scores indicating more severe rickets. The RSS provides an objective measure for monitoring disease progression, evaluating treatment effectiveness, and comparing clinical outcomes among patients [16].

### 2.3. Genetic Testing

Genomic DNA was extracted from whole-blood samples using the QIAamp DNA Blood Kit (Qiagen, Hilden, Germany). Genetic testing was conducted at GC Genome (GC Genome Corporation, Yongin, Republic of Korea) and Invitae (Invitae Corporation, San Francisco, CA, USA). At GC Genome, all exons of 5870 genes were captured using the Celemics G-Mendeliome DES Panel. Sequencing was performed on the MGI DNBSEQ-G400 platform, generating 2 × 100 bp paired-end reads. The DNA sequence reads were aligned to the reference sequence based on public human genome build GRCh37/UCSC hg19. At Invitae, a hypophosphatemia panel, including 13 genes (*ALPL* (NM_000478.5), *CLCN5* (NM_000084.4), *CYP27B1* (NM_000785.3), *CYP2R1* (NM_024514.4), *DMP1* (NM_004407.3), *ENPP1* (NM_006208.2), *FAH* (NM_000137.2), *FAM20C* (NM_020223.3), *FGF23* (NM_020638.2), *FGFR1* (NM_023110.2), *PHEX* (NM_000444.5), *SLC34A3* (NM_080877.2), and *VDR* (NM_001017535.1)) were used. Genomic DNA samples were enriched for targeted regions using a hybridization-based protocol and sequenced using Illumina technology, as previously described [18].

The data were filtered and analyzed to identify the sequence variants using an in-house bioinformatics pipeline. The identified variants were interpreted according to the American College of Medical Genetics and Genomics (ACMG) classification system [19] and cross-referenced with the ClinVar database to confirm the pathogenicity.

### 2.4. Statistical Analysis

A statistical analysis was conducted, using SPSS Statistics 20 to calculate the means and standard deviations. A *p*-value of <0.05 was considered statistically significant.

## 3. Results

### 3.1. Demographics and Clinical Presentation

**Figure 1:**
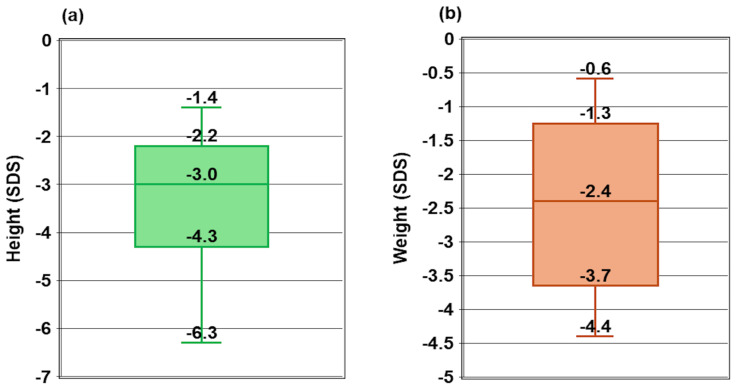
Height (**a**) and weight (**b**) at diagnosis.

The study included 19 children from 17 unrelated families, with a relatively balanced gender distribution: 52.6% were male, and 47.4% were female (Table 2). The median age of diagnosis of rickets was 19.2 months, ranging from 8.3 to 34.4 months. The median time of diagnosis of vitamin D-dependent rickets type 1A (VDDR1A) was 7.5 months, with a wide range from 1.1 to 148.0 months. The median height was −3.0 SDS, ranging from −6.3 SDS to −1.4 SDS (Figure 1a), while the median weight was −2.4 SDS, ranging from −4.4 SDS to −0.6 SDS (Figure 1b).

All children (100%) exhibited thickened wrists and ankles (Table 2). Genu varum or genu valgum was present in 94.7% of the children. Delayed walking was observed in 57.9% of the cases. Frontal bossing and chest deformities were noted in 52.6% of the children. Seizures occurred in 31.6% of the children, while bone fractures were reported in only two cases (10.5%). The average age for the eruption of the first primary tooth was 7.5 months, with the earliest at 6 months and the latest at 22 months. Delayed tooth eruption after 12 months was observed in 35.5% of the children. Fourteen out of nineteen children had yellowish enamel or fragile teeth.

**Figure 2:**
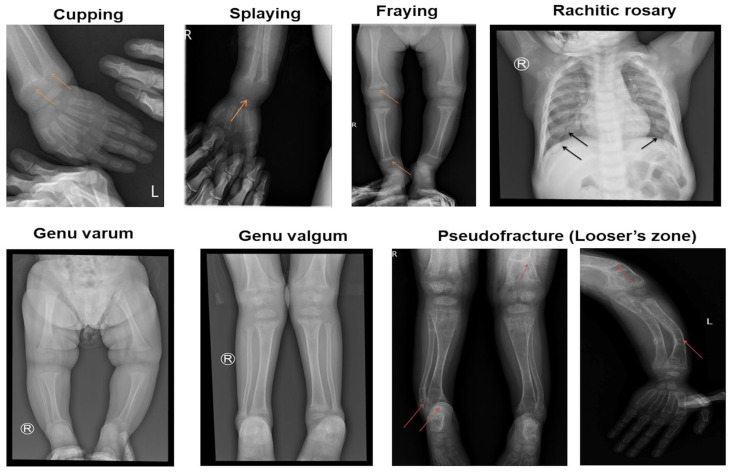
Abnormalities in radiologic images.

Cupping, splaying, and fraying were observed in all children in this study (Table 2 and Figure 2). Rachitic rosary was present in 12 out of 19 children (63.2%). Pseudofractures on the compression side of the bone, known as Looser’s zones, were observed in six out of nineteen children (31.6%). All children had a Rickets Severity Score (RSS) of 10, indicating severe rickets.

The serum total calcium levels were significantly lower than the normal reference range (2.2–2.6 mmol/L), with a mean value of 0.5 ± 0.3 mmol/L, indicating hypocalcemia (Table 3). The serum phosphate levels were also reduced (0.8 ± 0.4 mmol/L; normal: 1.05–1.9 mmol/L), with 11 out of 19 children identified as having hypophosphatemia. The alkaline phosphatase (ALP) levels were markedly elevated (1644.2 ± 917.1 UI/L; normal: 156–369 UI/L), reflecting increased bone turnover. The parathyroid hormone (PTH) levels were significantly elevated (457.7 ± 260.7 ng/mL; normal: 11–69 ng/mL), indicating secondary hyperparathyroidism. Despite these metabolic abnormalities, the 25-hydroxyvitamin D levels were within a broad range (140.5 ± 109.0 nmol/L; normal: 50–250 nmol/L).

### 3.2. Genetic Findings

**Figure 3:**
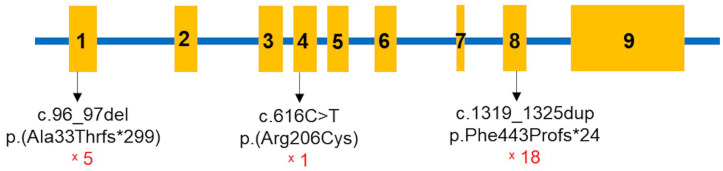
The *CYP27B1* mutation spots identified in 19 Vietnamese children with VDDR1A. The red font indicates the number of children harboring the variant.

All children in the study were found to have pathogenic variants in both alleles of the *CYP27B1* gene (Table 4, Figure 2). A total of three *CYP27B1* variants were identified among the 19 children: c.96_97del p.(Ala33Thrfs*299), c.616C>T p.(Arg206Cys), and c.1319_1325dup p.(Phe443Profs*24). The most common variant was c.1319_1325dup p.(Phe443Profs*24) in exon 8. Excluding patient 2, eighteen children carried this mutation, with fourteen in the homozygous state and four in the compound heterozygous state. The c.96_97del p.(Ala33Thrfs*299) variant was present in five children, while c.616C>T p.(Arg206Cys) was identified in one patient (Figure 3). Sanger sequencing was performed for the parents of 13 of the families, revealing that all were carriers of pathogenic variants in the heterozygous state (Table 4).

The c.1319_1325dup p.(Phe443Profs*24) variant was reported in dbSNP155, as well as in the ClinVar database, as a pathogenic variant. It has also been documented in VDDR1A patients in the previous literature (Table 5). The c.96_97del p.(Ala33Thrfs*299) variant was reported as pathogenic in the ClinVar database, but has not been described in the literature (Table 5). The c.616C>T p.(Arg206Cys) variant was not previously reported in ClinVar, dbSNP155, or the literature (Table 5). According to the ACMG guidelines, it was classified as a likely pathogenic variant (Table 5). No genotype–phenotype correlation was observed in this study.

## 4. Discussion

The median age of rickets diagnosis in this study was 19.2 months, with the youngest being 8.3 months and the oldest 34.4 months, consistent with findings from other studies [10,17,23]. Lin et al. reported a mean age of 2.1 ± 0.8 years in twelve children from so.uthern China with VDDR1A [23]. In Turkish patients, Dursun et al. observed a mean age at diagnosis of 13.1 ± 7.4 months among 11 children [10]. Similarly, Kaygusuz et al. found a median age of 16.0 months at diagnosis among 24 children with a homozygous p.Phe443Profs*24 genotype [17]. The delay in diagnosis reflects the rarity of the disorder and its nonspecific early symptoms. According to Haffner et al., the symptoms of rickets are most pronounced during infancy and puberty, stages characterized by increased calcium demands for growth [9]. In this study, the median time to a definitive VDDR1A diagnosis was 7.5 months. The earliest diagnosis was made concurrently with rickets detection, facilitated by an affected older sibling, whereas the latest diagnosis was made after twelve years. The primary reason for delayed diagnosis was the limited access to genetic analysis, which is essential for confirming VDDR1A.

Growth impairment was evident among the children in this study. The median weight standard deviation score (WtSDS) was −2.4, reflecting significant stunting and failure to thrive. At diagnosis, 18 out of 19 children had a height standard deviation score (HtSDS) below −2 SDS (WHO) compared to the normal reference range. The median HtSDS was −3.0 SDS, ranging from −6.3 to −1.4, consistent with the findings of Lin et al. [23], who reported a mean HtSDS of −3.8 ± 2.1 (range: −6.5 to −0.4). In contrast, Kaygusuz et al. [17] observed a slightly higher height at diagnosis, with a mean of −2.22 SD (range: −5.7 to −0.25). The growth impairment in this study was likely due to defective bone mineralization, particularly affecting the growth plates of the long bones [24].

All children in this study exhibited skeletal abnormalities, including enlargement of the wrists and ankles and lower limb deformities, indicating severe disruptions in bone mineralization and alignment. Other rickets-related symptoms, such as rachitic rosary and chest deformities, were less common. These findings are consistent with Lin et al., who reported a high prevalence of thickened wrists and ankles (91.7%), rachitic rosary (50.0%), and pectus carinatum (50.0%) [23]. Similarly, Ozden et al. reported that among nine children diagnosed with VDDR1A, four out of five had wrist and ankle enlargement, six out of nine exhibited rachitic rosary, and chest deformities were present in two out of nine cases [25]. In our study, enamel hypoplasia was observed in 73.7% of the children. This finding is comparable to Gjørup et al., who reported that five of six patients (83.3%) with VDDR1A had enamel hypoplasia [26].

Motor development was significantly delayed in 57.9% of the children, with delayed walking being the most common reason for seeking medical attention. This delay was mainly due to osteomalacia, which compromises bone structural integrity, hindering the ability to support weight and physical activity [9]. Additionally, seizures resulting from hypocalcemia were reported in six out of nineteen children (31.5%). Notably, seizures were relatively uncommon in the children under six months of age, reflecting an age-dependent variability in clinical presentation. These findings are consistent with Edouard et al., who reported hypocalcemic seizures in four out of twenty-one children with VDDR1A [12]. Similarly, in the study by Dursun et al., only one out of eleven children exhibited seizure symptoms [10].

In this study, only two children presented with bone fractures, which is lower than the incidence reported by Lin et al., who documented fractures in four out of twelve children [23]. It is also comparable to the findings of Tahir et al., who observed fractures in three out of twenty-two children [27]. This relatively low incidence of fractures contrasts with the findings from other studies, suggesting variability in the fracture occurrence among children with VDDR1A.

Laboratory evaluations revealed severe disruptions in the calcium–phosphate homeostasis and bone metabolism among the children. The mean total serum calcium was 1.5 ± 0.3 mmol/L, consistent with hypocalcemia. The serum phosphate levels were moderately low, with 11 children showing hypophosphatemia. These findings align with the typical biochemical profile of VDDR1A. In VDDR1A, serum phosphate levels are generally reduced due to the secondary hyperparathyroidism induced by hypocalcemia. Hypocalcemia triggers the increased secretion of parathyroid hormone (PTH), which enhances renal phosphate excretion, leading to further declines in the serum phosphate levels [28]. This pattern of hypophosphatemia was also reported in other studies of vitamin D-dependent rickets, as observed by Dursun et al. [10], Lin et al. [23], and Tahir et al. [27].

The Rickets Severity Score (RSS) of 10 observed in this study showed advanced rickets with significant skeletal deformities. This high score reflected severe clinical manifestations, including pronounced bowing of the long bones, widened growth plates, and metaphyseal fraying. These findings highlight the chronic and inadequately treated progression of the disease [16].

Radiographic abnormalities are crucial in the diagnostic assessment of rickets [9]. Cupping, splaying, and fraying, the characteristic radiographic features of rickets, were observed in all children in this study. Pseudofractures, known as Looser’s zones, were also identified. These pseudofractures occur due to the mechanical stress exerted by major blood vessels on the uncalcified cortices of osteomalacic bones, leading to symmetrical locations of transverse zones of rarefaction. These pseudofractures typically range from 1 mm to 1 cm in width, and are often multiple, symmetrically distributed, and can appear in otherwise structurally normal bones [29]. In this study, six children showed radiographic evidence of pseudofractures.

The c.1319_1325dup p.(Phe443Profs*24) variant, a common mutation found worldwide in VDDR1A patients [17], was also the most frequently identified variant in this study. The second most common variant was c.96_97del p.(Ala33Thrfs*299), which causes a frameshift at codon 33, leading to a premature stop codon after an additional 299 amino acids. This study is the first to report this pathogenic variant in a VDDR1A patient. A novel missense mutation, c.616C>T p.(Arg206Cys), was also identified. This mutation results in the substitution of arginine with cysteine at codon 206 of the *CYP27B1* protein. These nucleotide changes cause structural alterations in the *CYP27B1* protein, leading to abnormal protein function.

No genotype–phenotype correlation was observed in this study. There were no significant differences in the total blood calcium, ionized calcium, alkaline phosphatase (ALP), or parathyroid hormone (PTH) levels among children with different genotypes. These findings differ from those reported by Kaygusuz et al., who identified an association between the c.195+2T>G genotype and severe clinical phenotypes, as well as the p.K192E genotype and milder clinical manifestations [17]. In contrast, neither of these genotypes were present in the children in this study.

All children were treated with calcitriol and calcium and able to walk after 4.7 ± 1.5 months of treatment. The wrist and ankle enlargement resolved after 12 months of treatment. The total serum calcium, ALP, and PTH levels became normalized after 6 months of treatment. The Rickets Severity Scores (RSSs) showed marked improvement, with a median score of 1 (range: 0–3) after 12 months of treatment.

## 5. Conclusions

Vitamin D-dependent rickets type 1A is a rare disorder caused by pathogenic variants in the *CYP27B1* gene, leading to a loss of or reduction in 1α-hydroxylase activity, which impairs skeletal mineralization and causes bone deformities. The most common genotype identified in this study was the homozygous pathogenic variant c.1319_1325dup in exon 8. However, no clear association between genotype and phenotype was observed. Two novel *CYP27B1* variants were identified, expanding the known mutation spectrum of vitamin D-dependent rickets type 1A.

## Figures and Tables

**Table 1:** Rickets Severity Score table [16].

Evaluation Site	Grade	Radiographic Features
Radius and ulna	0	Normal
	1	Widened growth plate, irregularity of metaphyseal margins, no concave cupping
	2	Metaphyseal concavity with fraying of margins
Femur and tibia	0	Normal
	1	Partial lucency, smooth metaphyseal margin visible
	2	Partial lucency, smooth metaphyseal margin not visible
	3	Complete lucency, epiphysis appears widely separated from distal metaphysis
Multiplier	Multiplier 0.5	
	Multiplier 1	

**Table 2:** Clinical characteristics of children with VDDR1A.

Characteristics		Parameter *(n* = 19)
**Sex**	Male	10 (52.6%)
	Female	9 (47.4%)
**Age of diagnosis,** **rickets (month** **s** **)**		19.2 [8.3–34.4]
**Time of diagnosis,** **VDDR1A (month** **s** **)**		7.5 [1.1–148.0]
**Delayed walking**		11 (57.9%)
**Frontal bossing**		10 (52.6%)
**Thickened wrists and ankles**		19 (100%)
**Genu varum** **or** **genu valgum**		18 (94.7%)
**Rachitic rosary**		12 (63.2%)
**Bone fra** **c** **ture** **s**		2 (10.5%)
**Seizures**		6 (31.5%)
**Chest deformit** **ies**		10 (52.6%)
**Tooth eruption (month** **s** **)**		7.5 [6.0–22.0]
**Delayed tooth eruption**		6/17 (35.3%)
**Yellowish enamel or fragile teeth**		14/19 (73.7%)
**X-ray features**	Cupping and splaying	19 (100%)
	Fraying	19 (100%)
	Rachitic rosary	12 (63.2%)
	Pseudofracture (Looser’s zone)	6 (31.6%)
Rickets Severity Score = 10		19 (100%)

**Table 3:** Biochemical findings of children with VDDR1A.

Subclinical Testings	Normal Range	n	Results	Note
Total serum calcium (mmol/L)	2.2–2.6	19	1.5 ± 0.3	18 hypocalcemia
Serum phosphate (mmol/L)	1.05–1.95	19	0.8 ± 0.4	11 hypophosphatemia
Alkaline phosphatase (UI/L)	156–369	18	1644.2 ± 917.1	18 elevated
Parathyroid hormone (ng/L)	11–69	16	457.7 ± 260.7	16 elevated
25-hydroxyvitamin D (nmol/L)	50–250	17	140.5 ± 109.0	

**Table 4:** *CYP27B1* variants identified in Vietnamese children with VDDR1A.

Patient	Sex	Age of Onset (Months)	Age of Diagnosis (Months)	Exon	State in the Children	c.DNA Change	Protein Change	Inheritance
1	M	12.1	160.9	8	Homozygous	c.1319_1325dup	p.Phe443Profs*24	Maternal/Paternal
2a	M	24.9	43.0	8	Homozygous	c.1319_1325dup	p.Phe443Profs*24	Maternal/Paternal
2b	F	28.5	28.6	8	Homozygous	c.1319_1325dup	p.Phe443Profs*24	Maternal/Paternal
3	M	22.9	105.5	1/4	Compound heterozygous	c.96_97del/ c.616C>T	p.Ala33Thrfs*299/ p.Arg206Cys	n/a
4a	F	11.1	11.2	8	Homozygous	c.1319_1325dup	p.Phe443Profs*24	Maternal/Paternal
4b	M	17.5	70.4	8	Homozygous	c.1319_1325dup	p.Phe443Profs*24	Paternal/Maternal
5	F	19.3	21.8	8/1	Compound heterozygous	c.1319_1325dup/ c.96_97del	p.Phe443Profs*24/ p.Ala33Thrfs*299	Paternal/Maternal
6	F	31.4	50.0	8/1	Compound heterozygous	c.1319_1325dup/ c.96_97del	p.Phe443Profs*24/p.Ala33Thrfs*299	n/a
7	F	17.1	18.1	8	Homozygous	c.1319_1325dup	p.Phe443Profs*24	Maternal/Paternal
8	F	25.0	101.1	8	Homozygous	c.1319_1325dup	p.Phe443Profs*24	Maternal/Paternal
9	F	20.4	21.8	8	Homozygous	c.1319_1325dup	p.Phe443Profs*24	Maternal/Paternal
10	M	14.4	47.3	8	Homozygous	c.1319_1325dup	p.Phe443Profs*24	Maternal/Paternal
11	M	34.4	37.1	8/1	Compound heterozygous	c.1319_1325dup/ c.96_97del	p.Phe443Profs*24/p.Ala33Thrfs*299	Maternal/Paternal
12	F	15.1	15.7	8	Homozygous	c.1319_1325dup	p.Phe443Profs*24	n/a
13	M	25.2	110.0	8	Homozygous	c.1319_1325dup	p.Phe443Profs*24	Paternal/Maternal
14	M	13.5	14.6	8	Compound heterozygous	c.1319_1325dup/ c.96_97del	p.Phe443Profs*24/p.Ala33Thrfs*299	Maternal/Paternal
15	M	20.2	167.3	8	Homozygous	c.1319_1325dup	p.Phe443Profs*24	Maternal/Paternal
16	M	12.3	13.4	8	Homozygous	c.1319_1325dup	p.Phe443Profs*24	Maternal/Paternal
17	F	8.3	11.1	8	Homozygous	c.1319_1325dup	p.Phe443Profs*24	n/a

M: male, F: female, n/a: not analyzed.

**Table 5:** Classification of three *CYP27B1* variants identified in this study.

c.DNA Change	Aa Change	Effect	Mutation Taster	dbSNP155	ClinVar	ACMG Classification	Literature
c.96_97delAG	p.Ala33Thrfs*299	Frameshift	Disease causing	rs1955367513	Pathogenic	Pathogenic (PVS1, PM2, PM3, PP1, PP3, PP4, and PP5)	Novel
c.616C>T	p.Arg206Cys	Missense	Disease causing			Likely pathogenic (PM2, PM3, PP3, and PP4)	Novel
c.1319_1325dup	p.Phe443Profs*24	Frameshift	Disease causing	rs780950819	Pathogenic	Pathogenic (PVS1, PM2, PM3, PP3, PP4, and PP5)	[10,17,20,21,22]

## Data Availability

The original contributions presented in this study are included in the article. Further inquiries can be directed to the corresponding authors.
